# Evaluation of procalcitonin in patients with sepsis in Albanian adults

**DOI:** 10.1186/cc11803

**Published:** 2012-11-14

**Authors:** E Puca, P Pipero, A Pilaca, G Huti, A Daka, E Qyra, G Stroni, D Kraja, E Puca

**Affiliations:** 1 University Hospital Center 'Mother Teresa', Tirana, Albania; 2 American Hospital, Tirana, Albania

## Background

Sepsis is a systemic response to infection by microbial organisms [1]. However, differentiating sepsis from non-infectious triggers of the systemic inflammatory response syndrome (SIRS) is difficult. One promising marker is procalcitonin (PCT), whose concentration has been found to be elevated in sepsis [1-3]. The traditional clinical signs and the routine laboratory tests for sepsis, such as C-reactive protein (CRP) or leukocyte count, lack diagnostic accuracy and are sometimes misleading [1]. The use of biomarkers provides a novel, complementary approach to diagnose infection. In most infections, a true gold standard for diagnosis does not exist, thus the measurement of biomarkers, specifically the hormokine PCT in a clearly defined setting, has been shown to significantly improve the diagnostic certainty [3]. The aim of the study was to assess the value of white blood cell count (WBC), CRP, and PCT for the diagnosis of SIRS and sepsis. In Albania, PCT has been used as a serum marker for bacterial sepsis since May 2010.

## Methods

We evaluated WBC, CRP, and PCT in 143 adult patients presented to the Service of Infection Diseases, University Hospital Center 'Mother Teresa' and the American Hospital, Tirana, Albania. We used the Elycsys BRAHMS PCT test Electro Chemiluminescent Immunoassay (ECLIA) for the determination of PCT. We evaluated those parameters in three subgroups: 59 patients with sepsis, 64 patients with SIRS, and 20 patients as a control group. The cutoff of PCT for sepsis positivity was ≥2 ng/ml. Blood cultures were performed on the admission day or on the day of fever onset for the hospitalized patients.

## Results

In the sepsis subgroup the mean ± SD age was 53.4 ± 22.8 years. Average values of PCT, PCR and WBC were respectively 17.8 ng/ml, 37.3 ng/l and 13.7 × 10^3 ^cells/mm^3^. We excluded a case with septic shock with PCT value 761 ng/l. In SIRS subgroups the mean age was 46.5 ± 18.3 years and the average value of PCR and WBC were 21.3 ng/l and 11.6 × 10^3 ^cells/mm^3^, but PCT was ≤2 ng/ml. In the control group the mean age was 44.3 ± 25.24 years, and WBC, PCR and PCT were in normal values (respectively 3.5 to 10 × 10^3 ^cells/mm^3^, 0 to 5 ng/l and 0.5 to 2 ng/ml). Blood culture resulted positive in 40.6% of patients with sepsis.

## Conclusion

The serum concentration of PCT is specifically elevated in patients with sepsis. However, the evidence of PCT in infectious diseases is limited. The diagnostic model based on the laboratory parameters, using the combined predictors of PCT, CRP and WBC, can be a useful means for predicting early onset of sepsis.

## References

[B1] Jin M Khan AI Procalcitonin: uses in the clinical laboratory for the diagnosis of sepsis LABMEDICINE 2010 41 173 177

[B2] Kibe S Adams K Barlow G Diagnostic and prognostic biomarkers of sepsis in critical care J Antimicrob Chemother 2011 66 33 40 10.1093/jac/dkr121 21398306

[B3] Muñoz P Simarro N Rivera M Evaluation of procalcitonin as a marker of infection in a nonselected sample of febrile hospitalized patients Diagnostic Microbiol Infect Dis 2004 49 237 241 10.1016/j.diagmicrobio.2004.04.002 15313527

